# Old Mice Accumulate Activated Effector CD4 T Cells Refractory to Regulatory T Cell-Induced Immunosuppression

**DOI:** 10.3389/fimmu.2017.00283

**Published:** 2017-03-22

**Authors:** Idan Harpaz, Udayan Bhattacharya, Yehezqel Elyahu, Itai Strominger, Alon Monsonego

**Affiliations:** ^1^The Shraga Segal Department of Microbiology, Immunology and Genetics, Faculty of Health Sciences, Zlotowski Center for Neuroscience, The National Institute of Biotechnology in the Negev, Ben-Gurion University of the Negev, Beer Sheva, Israel

**Keywords:** aging, inflammation, CD4 T cells, senescence, regulatory T cells

## Abstract

Chronic low-grade inflammation and reduced lymphocyte potency are implicated in the pathogenesis of major illnesses associated with aging. Whether this immune phenotype results from a loss of cell-mediated regulation or intrinsic dysregulated function of effector T cells (Teffs) requires further research. Here, we report that, as compared with young C57BL6 mice, old mice show an increased frequency of CD4+CD62L− Teffs with a dysregulated activated phenotype and markedly increased effector functions. Analysis of the frequency and suppressive function of CD4+FoxP3+ regulatory T cells (Tregs) indicates an increase in the frequency of FoxP3+ T cells with aging which, however, occurs within the CD4+CD25− T cells. Furthermore, whereas Tregs from young and old mice similarly suppress Teffs from young mice, both have a compromised suppressive capacity of Teffs from old mice, a phenomenon which is partially recovered in the presence of IL-2-producing CD4+CD62L+ non-Teffs. Finally, we observed that Teff subsets from old mice are enriched with IL-17A-producing T cells and exhibit intrinsically dysregulated expression of genes encoding cell-surface molecules and transcription factors, which play a key role in T-cell activation and regulation. We, thus, demonstrate an age-related impairment in the regulation of effector CD4 T cells, which may underlie the higher risk for destructive inflammation associated with aging.

## Introduction

The significant deterioration of the immune system with age, which is known as immunosenescence and results from lifelong chronic antigenic stress (1, 2), increases the incidence and susceptibility to infection and degenerative diseases at older ages (3, 4). Paradoxically, although immunosenescence results in immune deficiency, it is also associated with chronic low-grade inflammation and with a higher risk of developing age-related diseases that usually, if not always, share an inflammatory pathogenesis (5, 6). The mechanism underlying this “Janus head” of immunosenescence is multidimensional and involves both the innate and adaptive immune compartments (7, 8).

One of the most remarkable changes that accompany the process of aging is related to alterations in the function and maintenance of CD4+ T cells (9–11). Although aging is associated with a reduced frequency of CD4+ T cells and with lymphopenia, it also shifts the phenotype of T cells from naïve to effector and memory phenotypes. In addition, human aging increases the frequency of activated CD4+ T cells (12), which, upon stimulation, express higher levels of pro-inflammatory cytokines [such as interferon-gamma (IFN-γ) (6, 13–15)]. The frequency of the highly pro-inflammatory Th17 T cells, which are implicated in several mouse models of autoimmune diseases (16–18), is also more prevalent in aged mice (19).

Despite intensive investigation of the frequency and competence of CD4+CD25highFoxP3+ regulatory T cells (Tregs) in aged humans (20–24) and mice (25–27), findings are still controversial; some studies reported no differences in the frequency and function of Tregs during aging (25, 26), whereas others show that the frequency and function of these cells are modified (20–23, 26, 27). Overall, whether the aberrant activation of effector T cells (Teffs) results primarily from intrinsic senescent properties of the cells or from a deficiency in Treg frequency and/or function is unclear. In this study, we, thus, analyze the changes Teff undergo with aging along with the frequency and function of Tregs.

## Materials and Methods

### Animals

All surgical and experimental procedures were approved by the Institutional Animal Care and Use committee of Ben-Gurion University of the Negev, Israel. C57BL/6 mice were purchased from The Jackson Laboratory (Bar Harbor, ME, USA) and were bred in our local specific pathogen-free animal facility. Mice were used at the age of 2–4 months (young mice) or at the age of 18–24 months (old mice).

### Flow Cytometry

We used a multicolor labeling technique to detect cell-surface and intracellular molecules in splenocytes. Briefly, spleens were removed from mice and dissociated in DMEM containing 10% fetal calf serum, 10 mM HEPES, 1 mM sodium pyruvate, 10 mM non-essential amino acids, 1% pen/strep, and 50 μL β-mercaptoethanol. ACK lysis buffer was added to the solution for 1 min to eliminate red blood cells. Viable mononuclear cells were counted in a hemocytometer by using trypan blue and adjusted to 1 × 10^6^ cells/mL. The following antibodies were used for cell-surface staining [all purchased from BioLegend (San Diego, CA, USA) or from Miltenyi biotec (Bergisch Gladbach, Germany)]: anti-CD25 (PE), anti-CD69 or anti-CCR6 (PeCy7), anti-CD62L (AF), and anti-CD4 or anti-CCR4 (Pacific Blue). To detect intracellular FoxP3, we used anti-FoxP3 (FITC or allophycocyanin) antibodies according to the manufacturer’s instructions (BioLegend). We collected data on a 10-color Gallios FACS machine and analyzed them with the Flowjo Pro software (FlowJo, LLC).

### Sorting

T cells were sorted using a two-step method. First, we enriched CD4+ T cells by using a CD4+ negative selection kit (BioLegend). Then, we stained CD4+ T cells with anti-CD25 (PE), anti-CD62L (APC), and anti-CD4 (PercP) and sorted them by FACS Aria cell sorter (BD biosciences, San Jose, CA, USA) to the following T-cell subsets: (1) non-Teffs: CD4+CD62L+ or CD4+CD62L+CD25−; (2) Teffs: CD4+CD62L−, CD4+CD62L−CD25−, CD4+CD62L−CD25−/low, or CD4+CD62L−CD25low, and (3) Tregs: CD4+CD25high.

### Stimulation Assay

Sorted CD4 T-cell subpopulations were stimulated with anti-CD3/anti-CD28 DynaBeads (Thermo Fisher scientific, MA, USA) for 48 h in U-shaped 96-well plates (0.5 × 10^5^ cells/well) using DMEM medium containing 10% fetal calf serum, 10 mM HEPES, 1 mM sodium pyruvate, 10 mM non-essential amino acids, 1% pen/strep, and 50 μL β-mercaptoethanol. For the Tregs suppression assay, we cocultured sorted Teff responders with sorted CD4+CD25high Tregs in a 2:1 ratio. After a 48 h incubation period, we harvested the supernatants and stored them at −80°C until cytokine analysis.

### Proliferation Assay

Sorted CD62L−CD25− effector CD4 T cells were labeled with 5 μM of carboxyfluorescein diacetate succinimidyl ester (CFSE) (Thermo Fisher Scientific, MA, USA) for 5 min in PBS containing 5% fetal calf serum at room temperature. Cells were then washed twice, cocultured with or without CD4+CD25high Tregs in a 2:1 ratio in U-shaped 96-well plates (0.5 × 10^5^ cells/well) and stimulated with anti-CD3/anti-CD28 DynaBeads. After a 60 h incubation period, CFSE dilution to daughter cells was analyzed with flow cytometry as previously described (28, 29).

### Measurements of Cytokine Production

We measured cytokine production (IL-2, IFN-γ, IL-10, and IL-17A) in supernatants with sandwich ELISA kits (BioLegend, San Diego, CA, USA), according to manufacturer’s instructions.

### RNA Extraction and Quantitative Reverse-Transcription PCR

We extracted total RNA from cell cultures with the miRNeasy Micro kit (Qiagen, Hilden, Germany) according to manufacturer’s instructions. We determined RNA concentrations by spectral absorbance at 260 nm and stored the specimens at −80°C. RNA (500 ng) was reverse transcribed with a high-capacity cDNA reverse transcription kit (Applied Biosystems, Carlsbad, CA, USA). Taq-Man Real-time PCR (Applied Biosystems and Roche, Basel, Switzerland) was used to quantify mRNA levels of RAR-related orphan receptor (RORγ), Tbet, GATA3, transforming growth factor beta receptor 1 (TGF-βR1), programmed cell death protein 1 (PD1), IL-10R, cytotoxic T-lymphocyte-associated protein 4 (CTLA-4), CD28, inducible T-cell costimulatory (ICOS), and FAS. All samples were assayed in triplicate. We used the glyceraldehyde 3-phosphate dehydrogenase gene as an endogenous control to normalize gene expression.

### Statistical Analyses

All statistical analyses were performed with GraphPad Prism, version 5.02, for windows (GraphPad software, San Diego, CA, USA). Data are presented as means ± SEM. *p* values were calculated with Student’s *t*-test or with two-way ANOVA.

## Results

### Aging Involves a Shift from Non-Effector to Effector CD4+ T-Cell Subsets

**Figure 1:**
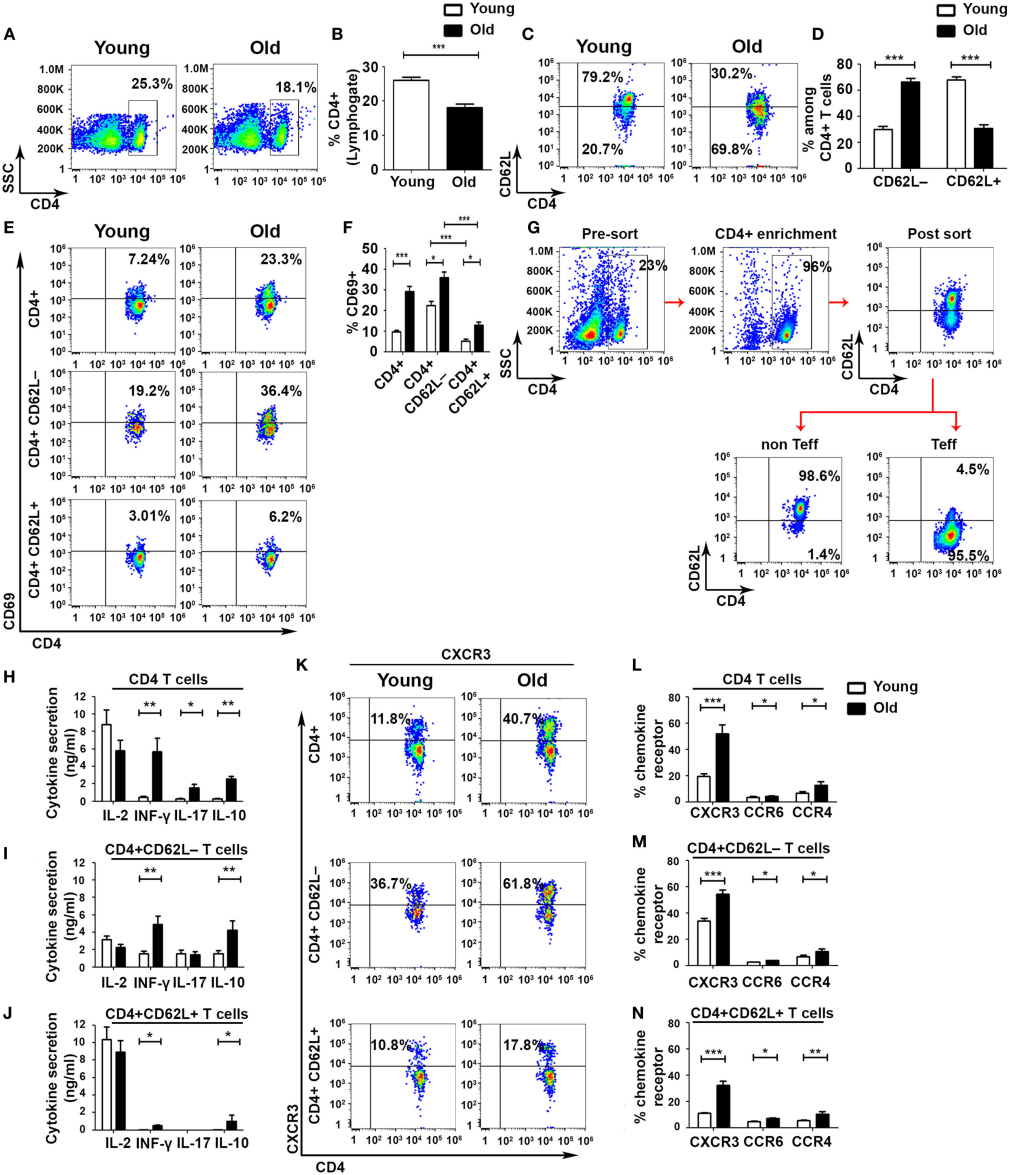
**Aging shifts the balance from non-effector into effector CD4+ T-cell subsets with enhanced effector functions**. Splenocytes were gated for lymphocytes **(A)** and then analyzed for the frequency of CD4+ T cells **(B)**. **(C–F)** CD4+ T cells were gated for CD4+CD62L− effector T cell (Teff) or for CD4+CD62L+ non-Teff subsets, and then the frequency of CD4+CD69+ T cells was analyzed. **(G–J)** Splenocytes were enriched for CD4+ T cells by magnetic separation, stained for CD4 and CD62L, sorted into CD4+CD62L+ and CD4+CD62L− T-cell subsets **(G)**, and stimulated with DynaBeads for 48 h. Then, cytokine secretion levels in the different CD4+ T-cell subsets were measured. **(K–N)** CD4+ T cells were gated for CD4+CD62L− Teff or for CD4+CD62L+ non-Teff subsets, and then the frequency of CXCR3, CCR6, and CCR4 chemokine receptors in the different subsets was analyzed. Data are shown as means ± SEM of 30 mice per group, pooled from five independent experiments. *p* values were calculated by Student’s *t*-test; *p < 0.05; **p < 0.01; ***p < 0.001.

To identify defects in cell-mediated immune regulation during aging, we divided CD4+ T cells into Teff and non-Teff subsets by using the L-selectin surface adhesion molecule CD62L, as described elsewhere (30). This analysis revealed a significant overall reduction in the frequency of CD4 T cells in old mice, as compared with young ones (Figures 1A,B), accompanied by a significant shift from a CD4+CD62L+ non-Teff subset to a CD4+CD62L– Teff subset in old mice (Figures 1C,D). The frequency of activated CD4+CD69+ T cells in both Teff and non-Teffs was increased in old mice, as compared with young ones (non-Teffs: 12.9 ± 1.64%, as compared with 5.28 ± 0.77%, respectively, *p* < 0.001; Teffs: 36 ± 2.72%, as compared with 22.5 ± 1.92%, respectively, *p* < 0.001; Figures 1E,F). In both young and old mice, the frequency of CD4+CD69+ T cells was significantly higher in the Teff subset than in the non-Teff subset (Figures 1E,F). To determine the functionality of Teff and non-Teff CD4+ T cells, we sorted CD4+CD62L+ non-Teff subsets and CD4+CD62L− Teff subsets obtained from young and old C57BL6 mice (Figure 1G) and then stimulated them with beads, as described in Section “Materials and Methods.” Stimulated CD4+ T cells obtained from old mice secreted significantly higher levels of the effector cytokines IFN-γ, IL-17A, and IL-10, as compared with the stimulated CD4+ T cells obtained from young mice (Figure 1H). In CD4+ T cells obtained from either young or old mice, stimulating the purified non-Teff and Teff subsets revealed that the Teff subset secreted lower levels of IL-2 and higher levels of the effector cytokines IFN-γ and IL-10, whereas the activated non-Teff CD4+ T cells secreted primarily IL-2 and very small amounts of effector cytokines (Figures 1I,J). In addition, the levels of secreted IFN-γ and IL-10 were significantly higher in the Teff subset of old mice than in the Teff of young ones (Figure 1I). An assessment of the expression of chemokine receptors revealed a predominant expression of the Th1 chemokine receptor CXCR3 in both young and old mice (Figures 1K–N). Within both the Teff and the non-Teff subsets, CD4+ T cells obtained from old mice exhibited an increased frequency of CXCR3+, CCR6+, and CCR4+ T cells, as compared with those obtained from young mice (Figures 1K–N and Figure S1 in Supplementary Material). Taken together, these data demonstrate that aging is accompanied by an increased incidence of activated Teffs and by a marked increase in cytokine release upon activation.

### The Frequency of FoxP3+ T Cells Is Increased among CD4+CD25− T Cells in Old Mice

**Figure 2:**
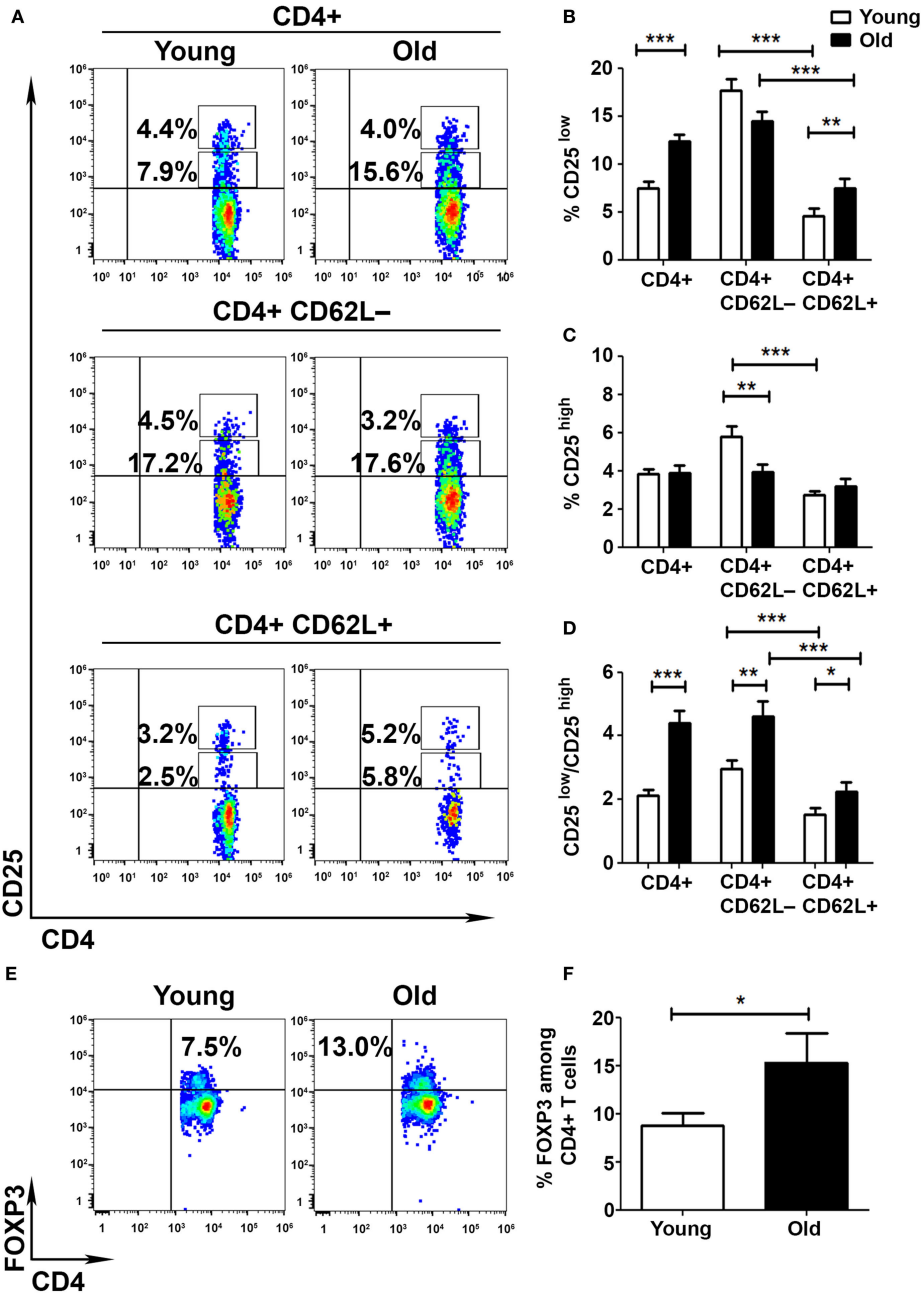

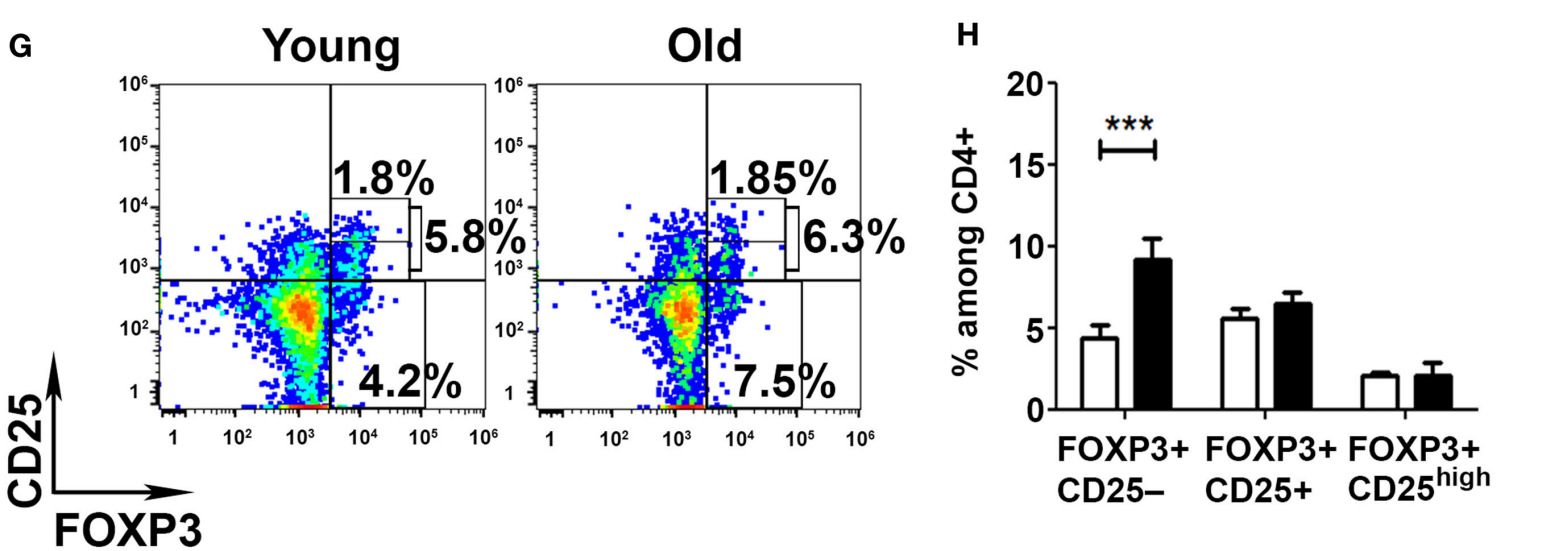
**Aging shifts the balance between activated and regulatory T cells (Tregs)**. To analyze the frequency of Tregs in young and old mice, splenocytes were harvested, stained for CD4, CD62L, CD25, CD127, and FoxP3, and subsequently analyzed by flow cytometry. **(A–D)** CD4+ T cells were gated for CD4+CD62L+ or CD4+CD62L− T cells **(A)**. Then, the frequency of CD4+CD25low **(B)** and CD4+CD25high **(C)** T cells was analyzed, and the activated/regulatory T-cell ratio among CD4+ T-cell subsets was calculated **(D)**. **(E,F)** CD4+ T cells were analyzed for the frequency of FoxP3+ T cells. **(G,H)** CD4+ T cells were analyzed for the frequency of FoxP3+CD25−, CD4+FoxP3+CD25+, and CD4+FoxP3+CD25high T cells. Data are shown as means ± SEM of 30 mice per group, pooled from three independent experiments. *p* values were calculated by Student’s *t*-test; *p < 0.05; **p < 0.01; ***p < 0.001.

To determine whether the aberrant activation of T cells obtained from old mice results from a deficient cell-mediated regulation, we pursued to analyze the frequency and function of Tregs in young and old mice. We initially assessed the frequencies of CD25low and CD25high subsets among CD4+ T cells obtained from young and old mice. FACS analysis revealed that CD4+ T cells are enriched with CD25low T cells in old mice, as compared with young mice (12.4 ± 0.7 and 7.5 ± 0.7%, respectively, *p* < 0.001; Figures 2A,B), and that this subset of cells is more abundant in the Teff (CD62L−) than in the non-Teff (CD62L+) compartment in both young and old mice (Figures 2A,B). In addition, whereas the frequency of CD4+CD25high T cells was higher in Teffs than in non-Teffs in young mice (5.8 ± 0.5 and 2.7 ± 0.2%, respectively, *p* < 0.001), it was comparable in both types of cells in old mice (Figures 2A,C). Accordingly, the Teff subset showed a significantly lower frequency of CD4+CD25high T cells in old mice, as compared with young mice (5.8 ± 0.5 and 4.0 ± 0.4%, respectively, *p* < 0.001; Figures 2A,C). The CD25low/CD25high ratio was thus significantly higher in old mice than in young mice, predominantly among Teffs (Figure 2D).

As a more accurate measurement of Treg frequency, we performed an intracellular staining of splenocytes with an anti-FoxP3. FACS analysis revealed that the frequency of CD4+FoxP3+ T cells was significantly higher in old mice than in young mice (15.4 ± 3.09 and 8.79 ± 1.24%, respectively, *p* < 0.05; Figures 2E,F). However, this increase in FoxP3+ T cells was evident only among the CD4+CD25− T-cell subsets (9.1 ± 1.35 and 4.4 ± 0.8%, respectively, *p* < 0.001; Figures 2G,H) and not among the CD4+CD25+ subsets. Overall, these data demonstrate that although the frequency of FoxP3+ T cells is increased in old mice, the frequencies of FoxP3+CD25+ and FoxP3+CD25high are similar in young and old mice. As previously shown (26, 31), the CD4+FoxP3+CD25− T cells that increase with aging may not exhibit the suppressive characteristics of CD4+FoxP3+CD25high T cells.

### Teffs of Old Mice Are Partially Resistant to Treg-Mediated Immunosuppression

**Figure 3:**
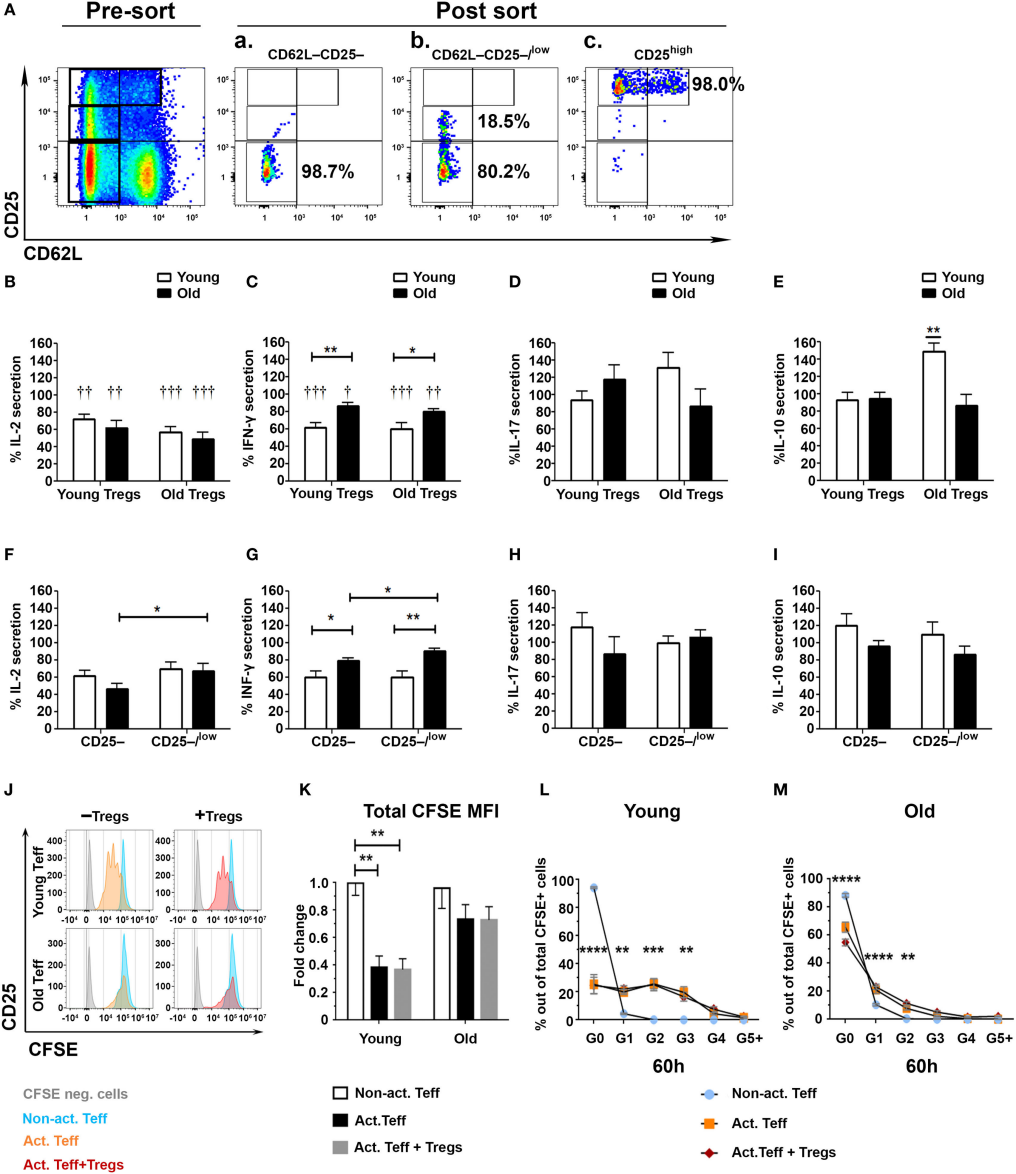
**Effector T cell (Teff) subsets from old mice are less susceptible to the immunosuppressive activity of regulatory T cells (Tregs)**. **(A)** Splenocytes were harvested from young and old mice, enriched for CD4+ T cells by magnetic separation, stained for CD4, CD62L, and CD25, and subsequently sorted into CD4+CD62L−CD25− **(Aa)** and CD4+CD62L−CD25−/low **(Ab)** Teff subsets or CD4+CD25high Tregs **(Ac)**. **(B–E)** CD4+CD25high Tregs from young or old mice were cocultured for 48 h with the CD4+CD62L−CD25− and with the CD4+CD62L−CD25−/low cell subsets at a 1:2 ratio, and then cytokine production was measured in the supernatants by ELISA. **(F–I)** CD4+CD25high Tregs from young and old mice were cocultured for 48 h with CD4+CD62L−CD25− or with CD4+CD62L−CD25−/low cell subsets at a 1:2 ratio, and then cytokine production was measured in the supernatants by ELISA. Data are shown as percentage of cytokine secretion in the presence of CD4+CD25high T cells [means ± SEM of 20 mice per group, pooled from four independent experiments (each with five mice per group)]. *p* values were calculated by Student’s *t*-test; *p < 0.05, ^†^*p* < 0.05. **(J–M)** Sorted carboxyfluorescein diacetate succinimidyl ester – (CFSE neg) CD4+CD25high Tregs from young or old mice were cocultured with CFSE-labeled CD4+CD62L−CD25− Teffs from young or old mice, respectively, in a 1:2 ratio. **(J)** Representative CFSE plots of young and old Teffs before and after stimulation (60 h). Teff proliferation was assessed by a fold decrease of CFSE MFI **(K)** and percent CFSE in each proliferation cycle as compared to non-activated (Non-act.) Teffs. Data are shown as means ± SEM of four biological repeats (each with 3–5 mice per group). *p* values were calculated by two-way ANOVA; **p < 0.01, ***p < 0.001, ***p < 0.0001.

Although the results in Figure 2 show no significant difference in the frequency of CD4+CD25high Tregs between young and old mice, they do show that aging causes a significant increase of effector CD4+CD25low T cells along with an increase in the CD25low/CD25high ratio. We, thus, pursued to determine the suppression capacity of sorted CD4+CD25high Tregs from young and old mice on both sorted CD4+CD62L−CD25− and CD4+CD62L−CD25−/low Teffs (Figure 3A). CD4+CD25high Tregs were first cultured with CD25− or with CD25−/low Teffs, in a 1:2 ratio and stimulated with anti-CD3/anti-CD28 DynaBeads. The secretion of IL-2 from Teffs of either young or old mice was suppressed to a similar extent by CD4+CD25high Tregs of young or old mice (Figure 3B). In addition, Tregs from either young or old mice suppressed the secretion of IFN-γ by Teffs; however, this immunosuppressive effect was significantly stronger on Teffs of young mice than on Teffs of old ones (Figure 3C). The levels of secreted IL-10 and IL-17A were not suppressed by Tregs obtained from young or old mice (Figures 3D,E). Comparing the activity of Tregs (isolated from both young and old mice) on CD25– to their activity on CD25–/low Teffs revealed that, in cells derived from old mice, the CD25–/low Teff subset was more resistant than the CD25– subset to Treg-mediated suppression of IL-2 and IFN-γ (Figures 3F,G). In contrast, no differences were observed in Treg-mediated suppression of IL-17A and IL-10 among CD25− subset, as compared with the CD25−/low subset, in either young or old mice (Figures 3H,I).

To determine whether the reduced suppressive function of Tregs on Teffs from old mice results from enhanced proliferation of the Teffs or their enhanced cytokine production, we labeled sorted Teffs from young and old mice with CFSE and then cocultured them with Tregs from young or old mice, respectively. Figures 3J–M demonstrate that stimulated CD62L–CD25– Teffs from young mice underwent a similar number of proliferation cycles (Figures 3J,L) along with a similar reduction in CFSE MFI (Figure 3K) in the absence or in the presence of Tregs, whereas stimulation of this Teff subset from old mice yielded neither significant proliferation (Figures 3J,M) nor reduction in CFSE MFI (Figure 3K). These results suggest that Treg-mediated suppression of this particular Teff subset (CD4+CD25−CD62L−) is at the cytokine level and that this Teff subset from old mice not only exhibits dysregulated cytokine production but also fails to proliferate properly following stimulation.

**Figure 4:**
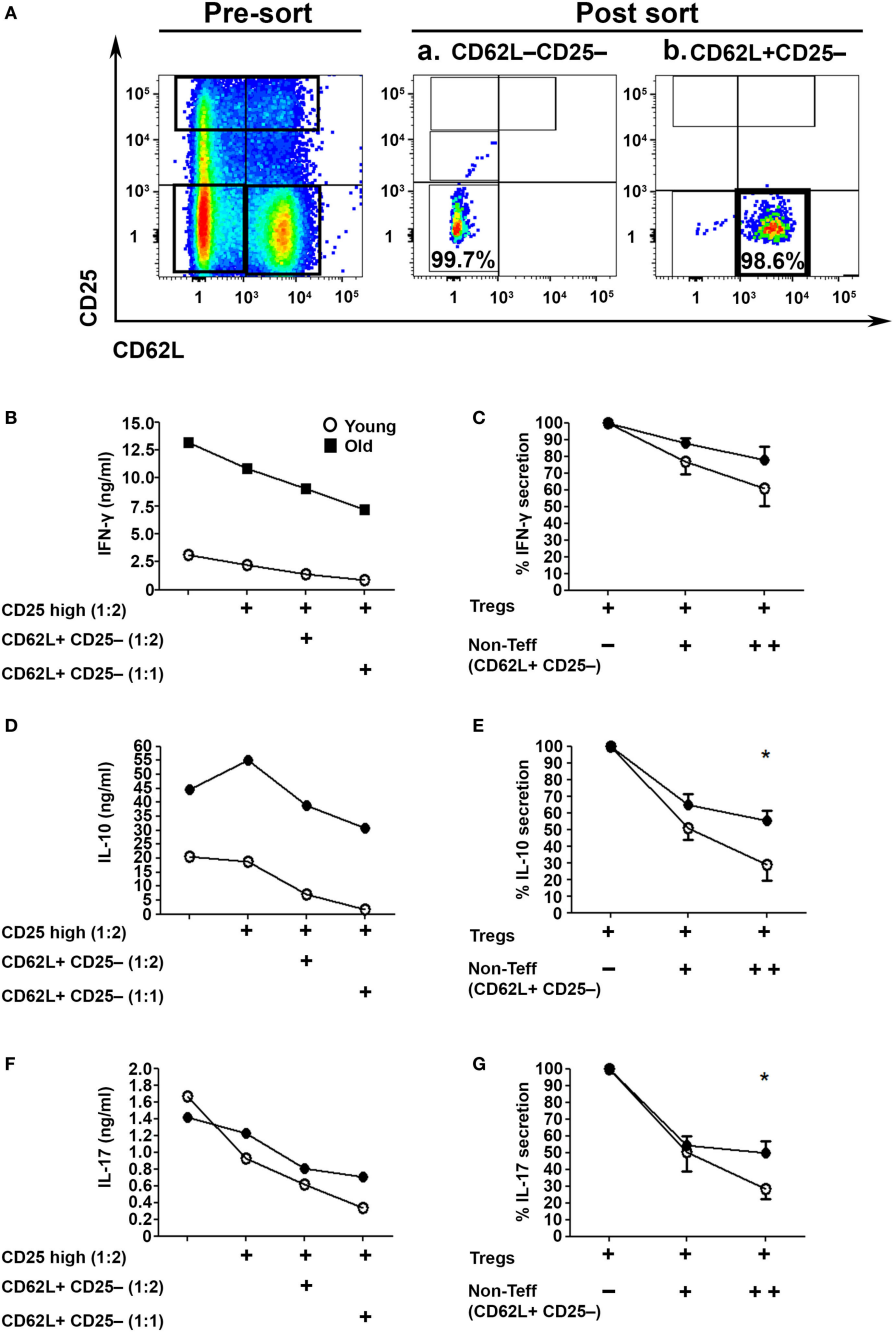
**CD4+CD62L+ non-effector T cell (Teff) subsets improve the immunosuppressive effects of CD4+CD25high Tregs in an age-dependent manner**. **(A)** Splenocytes were harvested from young and old mice, enriched for CD4+ T cells by magnetic separation, stained for CD4, CD62L, and CD25, and subsequently sorted into a CD4+CD62L−CD25− Teff subset **(Aa)**, a CD4+CD62L+CD25– non-Teff subset **(Ab)** or CD4+CD25high Treg cells as shown in Figure 3A. The sorted CD4+CD62L−CD25− Teff subsets were cocultured for 48 h with CD4+CD25high Treg cells at a2:1 ratio, with or without sorted CD4+CD62L+CD25− non-Teff subsets, and then cytokine production was measured in the supernatant by ELISA. **(B,D,F)** Cytokine secretion levels. **(C,E,G)** Percent cytokine secretion in the presence of CD4+CD62L+CD25− non-Teffs. Data are shown as means ± SEM of eight mice per group, pooled from two independent experiments (each with four mice per group). *p* values were calculated by Student’s *t*-test; *p < 0.05.

Since we examined the activity of Tregs in the absence of the CD4+CD62L+ subset, which is the major source of IL-2 within CD4+CD25− T cells (Figure 1J), we next examined their role in the Treg suppression assay (32). To this end, we sorted CD62L−CD25− Teffs (Figure 4Aa) and cocultured them with sorted CD4+CD25high Tregs in the presence or absence of CD4+CD25−CD62L+ non-Teff T cells (Figure 4Ab). The T cells were stimulated with anti-CD3/anti-CD28 DynaBeads for 48 h, and IFN-γ, IL-17A, and IL–10 cytokine secretion was measured with ELISA. The CD4+CD62L+ T cells significantly enhanced, in a dose-dependent manner, the suppression function of Tregs derived from either young or old mice (Figures 4B–G). Notably, however, CD4+CD62L–CD25– Teffs cells derived from old mice, as compared with those derived from young ones, secreted higher amounts of IFN-γ, IL-17A, and IL-10 (Figures 4B,D,F) and were less sensitive to the regulatory impact of CD4+CD62L+ T cells (Figures 4C,E,G).

### Teffs from Old Mice Exhibit Intrinsic Dysregulated Properties

**Figure 5:**
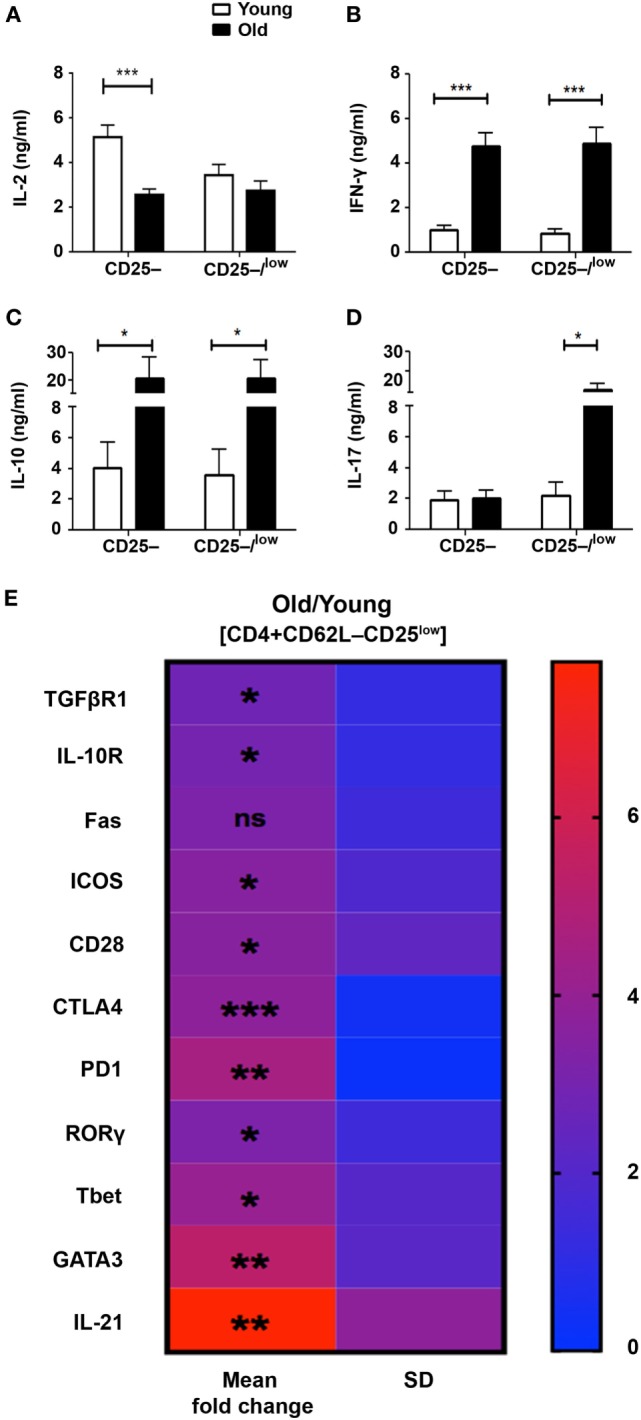
**The pattern of cytokine and gene expression is dysregulated in effector T cells (Teffs) from old mice**. **(A–D)** Splenocytes were harvested from young and old mice, enriched for CD4+ T cells by magnetic separation, stained for CD4, CD62L, and CD25, and subsequently sorted into CD4+CD62L–CD25– and CD4+CD62L–CD25–/low Teff subsets as shown in Figure 3A. The sorted Teff subsets were stimulated for 48 h and then cytokine production was measured in the supernatant by ELISA. Data are shown as means ± SEM of four or nine independent experiments (each with 4–5 mice per group) with CD4+CD62L–CD25–/low or CD4+CD62L–CD25– Teff subsets, respectively. **(E)** CD4+CD62L–CD25low Teff subsets were sorted from young and old mice and total RNA was extracted. The RNA was reverse transcribed with a high-capacity cDNA reverse-transcription kit, and gene expression was analyzed with qPCR and is shown as fold change in old mice relative to young mice. Data are shown as means ± SD of 3–4 mice per group, representing three independent experiments (each with 3–4 mice per group). *p* values were calculated by Student’s *t*-test; *p < 0.05; **p < 0.01; ***p < 0.001. The calculated means, SDs, and *p* values are provided in Table S1 in Supplementary Material.

Although the CD62L+ subset partially abrogated the resistance of Teffs from old mice to Treg suppression, the release of effector cytokines following activation remained substantially high. This finding indicates that Teffs from old mice exhibit intrinsic properties that attenuate the function of Tregs, and that this phenomenon is more pronounced in the CD25low Teff subset than in the CD25− Teff subset. We thus sorted both CD4+CD62L−CD25– and CD4+CD62L−CD25−/low Teff subsets and compared their cytokine profile following anti-CD3/anti-CD28 DynaBeads stimulation for 48 h. As compared with young mice, CD25− and CD25−/low Teffs derived from old mice tended to secrete lower levels of IL-2 (Figure 5A), but they secreted significantly higher levels of IFN-γ and IL-10 (Figures 5B,C). Furthermore, the secreted levels of IL-17 in the CD25– Teff subset were similar in cultures derived from both old and young mice, whereas they were markedly higher in CD25–/low Teff cultures derived from old mice (Figure 5D). Based on these data, we sorted the CD4+CD25low Teffs (excluding the CD25− subset) from young and old mice and analyze them by qRT-PCR. As shown in Figure 5E and Table S1 in Supplementary Material, mRNA levels of receptors that convey either stimulatory (e.g., CD28 and ICOS) or inhibitory/apoptotic (e.g., CTLA-4, TGF-βR1, IL-10R, PD1, and FAS) signals were higher in CD25low Teffs derived from old mice. Along with these data, the mRNA levels of the key polarizing transcription factors Tbet, RORγ, and GATA3 were significantly higher in CD25low Teffs from old mice (Figure 5E; Table S1 in Supplementary Material). Notably, IL-21, which is part of the differentiation and function of Th17 cells (17), was about seven-fold higher in this subset (Figure 5E; Table S1 in Supplementary Material).

## Discussion

The goal of this study was to elucidate mechanisms contributing to age-related chronic low-grade inflammation. Our results demonstrate that aging is accompanied by quantitative and qualitative impairments in effector CD4+ T cells. The Teff subsets from old mice exhibit an activated phenotype and are resistant to Treg-mediated immunosuppression—a defect that can be partially restored by IL-2-secreting non-Teffs. Finally, the Teff subsets from old mice are enriched with IL-17A-producing T cells and demonstrate intrinsically dysregulated expression of genes encoding cell-surface molecules and transcription factors which play a key role in T-cell activation and regulation. Our study thus suggests that aging accompanies a primary defect in CD62L– effector CD4 T cells which may prone to declined immunity and chronic inflammation.

An increased effector:naïve T-cells ratio was previously observed in older mice (33, 34) and humans (35, 36), but the molecular properties of the distinct Teff subsets that contribute to compromised immunity and chronic inflammation in old age are still unknown. By sorting the Teff and non-Teff CD4 subsets, we show that, effector cytokines are expressed primarily by CD62L− Teffs, whereas primarily IL-2 is expressed by the non-Teff CD62L+ cells. Such a distinction between the CD62L– and CD62L+ subsets allows an accurate analysis of the effector and regulatory properties of lymph node and inflammatory site-homing CD4 T cells. Focusing on the Teff subsets to elucidate the mechanisms underlying chronic inflammation in old age reveals that aging is accompanied by an increased frequency of readily activated CD4 Teffs. Following stimulation, Teffs from old mice secrete considerably higher levels of effector cytokines than Teffs from young mice, as was previously described in pathological conditions associated with chronic inflammation, e.g., in patients with Guillain–Barre syndrome, neuropathic diseases (37), and rheumatoid arthritis (38). Since the non-Teff subsets almost completely lack effector functions [(30, 33), Figure 1], the increased level of cytokine secretion from CD4+ T cells derived from old mice is most likely the result of a combination between an increased frequency of Teff subsets and an aberrant regulation of their function.

We then investigated whether cell-mediated regulatory mechanisms are impaired in old mice. We show that aging is accompanied by an imbalance between CD4+CD25low and CD4+CD25high T cells, predominantly among Teffs. This finding supports previous studies, which demonstrated that aging is associated with a shift from Tregs to Teff subsets (39). Also in line with previous studies, we show that the frequency of FoxP3+ T cells is increased in old mice (26, 40–43). However, this increase occurred within the CD4+CD25– and not within the CD4+CD25high subset. Previous studies have shown that mice lacking CD25 or the IL-2 cytokine demonstrate a similar increase in the number of immature CD4+CD25–FoxP3low Tregs (44–46) and have reduced capability to suppress autoreactive T cells *in vivo* (44, 47). Furthermore, CD4+CD25lowFoxP3+ T cells were recently implicated in the pathogenesis of RA as cells which can lose FoxP3 expression and accumulate at the inflamed joints as Th17 T cells (48). Taken together, our results demonstrate that, although the frequency of CD4+FoxP3+ T cells generally increased with aging, it occurred in our study within the CD4+CD25– subset which exhibited substantially increased effector functions as compared to this subset from young mice (Figure 5). Additional studies are though required to elucidate aging-related alterations in the frequency and functionality of CD4+FoxP3+CD25low/– T cells.

Beside the frequency, the functionality of Tregs is a crucial factor that contributes to immune tolerance (49, 50). CD4+CD25high Tregs from either young or old mice demonstrated, overall, similar suppressive functions when they were cocultured with CD4+CD62L−CD25− Teffs. This finding is in line with previous studies, which indicated that Tregs from young and old mice similarly suppress CD4 T-cell proliferation (23, 26, 41), IFN-γ production (40), and T cell-driven autoimmune colitis (25). An important aspect of our study relates to the capacity of Tregs to suppress old mice-derived Teffs: the CD4+CD62L−CD25−, and to a further extent, the CD4+CD62L−CD25−/low Teffs were resistant to Treg suppression as compared with the same T-cell subsets from young mice. Notably, Tregs did not affect the proliferation of these effector subsets but rather they suppressed the production of effector cytokines. Thus, given the marked higher levels of cytokines produced by Teffs from old mice, in particular, the IL-17A and IL-21 cytokines of the Th17 lineage, it is likely that the Treg resistance among Teff subsets from old mice is at the level of dysregulated cytokine production. Suppression of Teffs in our study was though substantially enhanced when, in addition to the Teff responder cells, the culture also included non-Teffs (which are a major source for IL-2), a phenomenon that was more pronounced in T-cell cultures derived from young mice than those derived from old mice. The implications of these findings are two-fold: first, rather than using the entire CD4+CD25− T cells (including effector and non-Teffs) as responder cells, the use of sorted resting (CD4+CD62L−CD25−) and activated (CD4+CD62L−CD25+) Teff subsets with or without the CD4+CD62L+CD25− non-Teffs, may provide a more accurate measure of the actual regulatory capacity of Tregs at inflammatory sites *in vivo*; second, while Tregs from old mice maintain their functional properties, they may fail to control chronic inflammation due to, at least in part, insufficient levels of IL-2 (32, 51–54). Further studies are required to determine the role of CD4+CD62L+ T cells in enhancing the suppression of Teffs both *in vitro* and *in vivo* along with the contribution of aging to this phenomenon.

Despite the comparable suppressive functions of Tregs from young and old mice, we found that Teffs from old mice are more resistant than Teffs from young mice to the immunosuppressive effects of Tregs. Such resistance to suppression was previously reported in several animal models of autoimmune diseases—including models of diabetes (55), experimental autoimmune encephalomyelitis (56), and systemic lupus erythematosus (57) and in humans with systemic lupus erythematosus (58, 59). Moreover, it has been shown that CD4+CD28null T cells, which accumulate during aging and are highly enriched with activated Teffs, are less prone to regulation by CD4+CD25high T cells, as compared with CD4+CD28+ T cells (60). In our study, this resistance was more pronounced when, in addition to the CD4+CD62L–CD25– T cells, the responder cells also included the CD25low Teff subset. An *ex vivo* assessment of the functionality and molecular signature of the CD4+CD25low Teff subset from old mice revealed that, as compared with young mice, this subset exhibits increased effector functions along with upregulation of the key polarizing transcription factors Tbet, RORγ, and GATA3 as well as upregulation of cell-surface molecules which tightly regulate T-cell activation. Taken together, our data suggest that aging results in accumulation of aberrantly regulated CD4 T cells which on one hand exhibit increased activation threshold (i.e., increased levels of CTLA-4 and PD1) and thus reduced potency and on the other hand dysregulated effector functions, which can cause chronic inflammation (7, 19, 40, 61, 62). A more extensive analysis is required to explore the changes Teff subsets undergo during aging along with identifying key regulators of this process possibly serving as therapeutic targets for intervention in T cell senescence.

In summary, the findings reported in this study highlight fundamental aging-related alterations of Teffs, which impair the balance between protective and pathogenic immune responses, and may explain the development of age-related chronic inflammation and/or a compromised immunity to pathogens, tumors, and tissue repair. Translated clinically, the ability of Treg cells to downregulate autoinflammation suggests that improving Treg functionality in old subjects via the administration of IL-2 or IL-2-producing T cells may have therapeutic implications for dampening the risk for senescence-associated inflammation without compromising immunity. Such a strategy may show better efficacy when, in parallel to IL-2 therapy, drugs targeting the intrinsic dysregulated properties of Teffs are explored.

## Author Contributions

IH designed and performed research, analyzed data and wrote manuscript. UB, YE, and IS performed research and analyzed data. AM designed research and wrote manuscript.

## Conflict of Interest Statement

The authors declare that the research was conducted in the absence of any commercial or financial relationships that could be construed as a potential conflict of interest.

## Abbreviations

Teffs, effector T cells; Tregs, regulatory T cells; IFN-γ, interferon gamma; RORγ, RAR-related orphan receptor; TGF-βR1, transforming growth factor beta receptor 1; PD1, programmed cell death protein 1; CTLA-4, cytotoxic T-lymphocyte-associated protein 4; ICOS, inducible T-cell costimulatory; GAPDH, glyceraldehyde 3-phosphate dehydrogenase.
