# Supplementary material for: Old Mice Accumulate Activated Effector CD4 T Cells Refractory to Regulatory T Cell-Induced Immunosuppression
Source: Front Immunol. 2017 Mar 22;8:283. doi: 10.3389/fimmu.2017.00283 (PMC5360761; doi:10.3389/fimmu.2017.00283)
Supplement: Supplementary file 1 [file image_1.pdf]

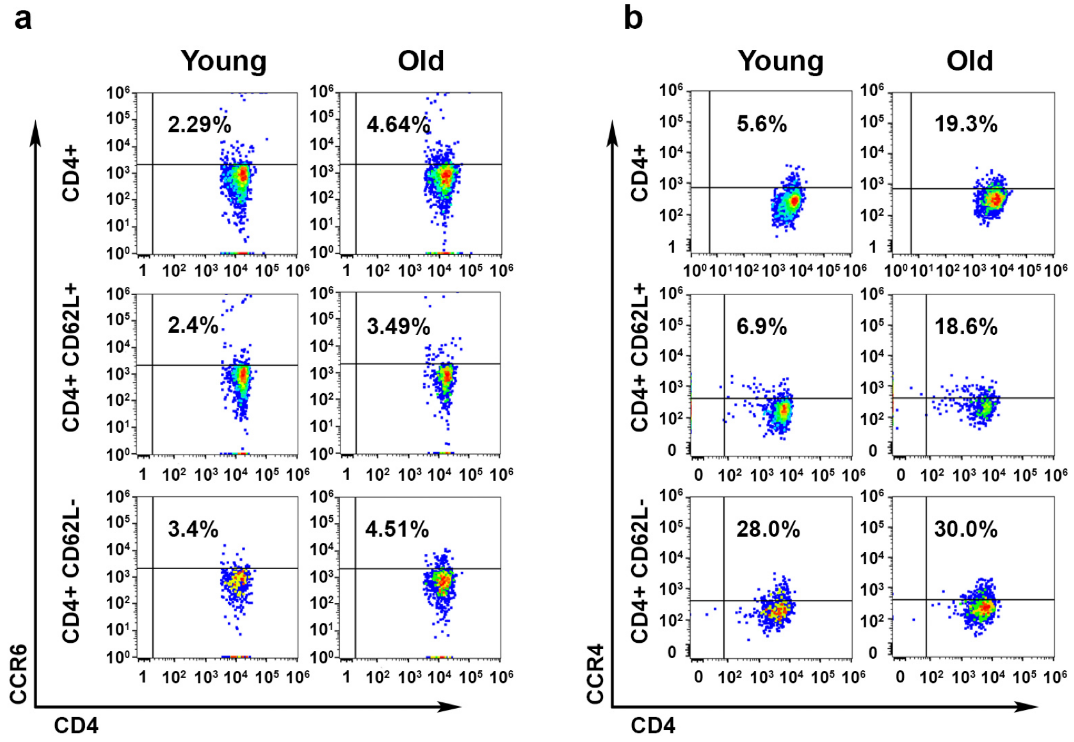

Supplemental Figure 1. Aging is associated with increased frequency of CD4 T cells expressing the chemokine receptors CCR6 and CCR4.

CD4+ T cells were gated for CD4+CD62L– Teff or for CD4+CD62L+ non-Teff subsets, and then the frequency of CCR6 (a) and CCR4 (b) chemokine receptors in the different subsets was analyzed. Data are shown in Fig. 1 as means ± SEM of 30 mice per group, pooled from five independent experiments. *p*-values were calculated by Student's *t*-test; *$p < 0.05$; **$p < 0.01$; ***$p < 0.001$
