# Supplementary material for: Old Mice Accumulate Activated Effector CD4 T Cells Refractory to Regulatory T Cell-Induced Immunosuppression
Source: Front Immunol. 2017 Mar 22;8:283. doi: 10.3389/fimmu.2017.00283 (PMC5360761; doi:10.3389/fimmu.2017.00283)
Supplement: Supplementary file 2 [file table_1.docx]

|  | **Mean** | SD | **P value** | Significance |
| --- | --- | --- | --- | --- |
| TGFβR | 2.791 | 1.122 | 0.0125 | ***** |
| IL-10R | 2.981 | 1.158 | 0.0105 | ***** |
| Fas | 3.204 | 1.426 | 0.1114 | **NS** |
| ICOS | 3.495 | 1.87 | 0.0187 | ***** |
| **CD28** | 3.534 | 2.310 | 0.0126 | ***** |
| CTLA4 | 3.723 | 0.392 | 0.0002 | ******* |
| PD1 | 4.540 | 0.160 | 0.0013 | ****** |
| RORγ | 3.206 | 1.427 | 0.0248 | ***** |
| Tbet | 4.059 | 2.035 | 0.0130 | ***** |
| GATA3 | 5.296 | 2.154 | 0.0099 | ****** |
| IL-21 | 7.749 | 3.684 | 0.0043 | ****** |

**Supplementary Table 1: The pattern of cytokine and gene expression is dysregulated in Teff cells from old mice.** CD4+CD62L–CD25^low^ Teff subsets were sorted from young and old mice and total RNA was extracted. The RNA was reverse-transcribed with a high-capacity cDNA reverse transcription kit, and gene expression was analyzed with qPCR and is shown as fold change in old mice relative to young mice. Data are shown as means ± SD of 3-4 mice per group, representing three independent experiments (each with 3-4 mice per group). *p*-values were calculated by Student’s *t*-test; **p <* 0.05; ***p <* 0.01; ****p <* 0.001.
